# The Effects of Super-Flux (High Performance) Dialyzer on Plasma Glycosylated Pro-B-Type Natriuretic Peptide (proBNP) and Glycosylated N-Terminal proBNP in End-Stage Renal Disease Patients on Dialysis

**DOI:** 10.1371/journal.pone.0092314

**Published:** 2014-03-25

**Authors:** Yasuaki Nakagawa, Toshio Nishikimi, Koichiro Kuwahara, Shinji Yasuno, Hideyuki Kinoshita, Yoshihiro Kuwabara, Kazuhiro Nakao, Takeya Minami, Chinatsu Yamada, Kenji Ueshima, Yoshihiro Ikeda, Hiroyuki Okamoto, Kazukiyo Horii, Kiyoshi Nagata, Kenji Kangawa, Naoto Minamino, Kazuwa Nakao

**Affiliations:** 1 Department of Medicine and Clinical Science, Kyoto University Graduate School of Medicine, Kyoto, Japan; 2 Department of EBM Research, Kyoto University Hospital, Institute for Advancement of Clinical and Translational Science, Kyoto, Japan; 3 Medical corporation, Ikeda Clinic, Kyoto, Japan; 4 Diagnostics Division, Shionogi & Co., Ltd, Oska, Japan; 5 Department of Biochemistry, National Cerebral and Cardiovascular Center Research Institute, Fujishirodai, Suita, Osaka, Japan; 6 Department of Molecular Pharmacology, National Cerebral and Cardiovascular Center Research Institute, Fujishirodai, Suita, Osaka, Japan; University of Sao Paulo Medical School, Brazil

## Abstract

**Background:**

Plasma BNP levels are predictive of prognosis in hemodialysis patients. However, recent studies showed that the current BNP immunoassay cross-reacts with glycosylated proBNP, and the NT-proBNP assay underestimates glycosylated NT-proBNP. In addition, the recently developed high performance dialyzer removes medium-sized molecular solutes such as β2-microgloburin. We therefore investigated the effects of high performance dialysis on measured levels of glycosylated proBNP, glycosylated NT-proBNP and other BNP-related peptides in end-stage renal disease (ESRD) patients on hemodialysis.

**Method:**

The relationships between clinical parameters and BNP-related molecule were also investigated. We used our newly developed immunoassay to measure plasma total BNP and proBNP in 105 normal subjects and 36 ESRD patients before and after hemodialysis. Plasma NT-proBNP was measured using Elecsys II after treatment with or without deglycosylating enzymes. We also measured plasma ANP and cGMP using radioimmunoassays.

**Results:**

All the measured BNP-related peptides were significantly higher in ESRD patients than healthy subjects. Total BNP (−38.9%), proBNP (−29.7%), glycoNT-proBNP (−45.5%), nonglycoNT-proBNP (−53.4%), ANP (−50.4%) and cGMP (−72.1%) were all significantly reduced after hemodialysis, and the magnitude of the reduction appeared molecular weight- dependent. Both the proBNP/total BNP and glycoNT-proBNP/nonglycoNT-proBNP ratios were increased after hemodialysis. The former correlated positively with hemodialysis vintage and negatively with systolic blood pressure, while the latter correlated positively with parathyroid hormone levels.

**Conclusion:**

These results suggest that hemodialysis using super-flux dialyzer removes BNP-related peptides in a nearly molecular weight-dependent manner. The ProBNP/total BNP and glycoNT-proBNP/nonglycoNT-proBNP ratios appear to be influenced by hemodialysis-related parameters in ESRD patients on hemodialysis.

## Introduction

Hemodialysis patients exhibit a greatly heightened risk of cardiovascular morbidity and mortality. For example, these patients experience an extremely high prevalence of left ventricular hypertrophy and heart failure. Consequently, there is a great need for a good clinical biomarker enabling early identification of dialysis patients at risk of cardiovascular events and mortality, as well as earlier aggressive intervention.

B-type natriuretic peptide (BNP; also termed brain natriuretic peptide) is a cardiac hormone produced and secreted mainly by the ventricles; another cardiac hormone, atrial natriuretic peptide (ANP), is produced and secreted by the atria. [1] Ventricular wall stress and/or ischemia stimulate expression of the BNP precursor proBNP [2], [3], which is thought to be cleaved to BNP-32 (BNP or mature BNP) and N-terminal proBNP (NT-proBNP) prior to its secretion [4]. Plasma levels of BNP, NT-proBNP and ANP are elevated in patients with cardiac hypertrophy or heart failure. [5] They are also elevated in patients with chronic kidney disease, especially those receiving dialysis. [6] This is likely related to left ventricular dysfunction as well as to reduced clearance and increased plasma volume. It is widely recognized that cardiac function is a major confounder influencing levels of these peptides in dialysis patients. Thus, even in dialysis patients with end-stage renal disease (ESRD), BNP is used for diagnosis and evaluation of the severity of heart failure and is predictive of patient prognosis. [7].

Recent studies have shown that unprocessed precursor proBNP circulates in healthy individuals [8], and that its levels in plasma are increased in patients with severe heart failure. This is noteworthy in part because the immunoassay system currently being used to measure BNP also detects proBNP (the anti-BNP antibody cross-reacts with proBNP). In fact, it appears that about 70% of the plasma BNP measured using the BNP immunoassay system is proBNP in healthy human subjects. [9] In addition, it was recently shown that recombinant proBNP derived from mammalian cells has seven sites capable of *O*-linked oligosaccharide attachment within the N-terminal portion of the peptide, [10] and that both proBNP and NT-proBNP are glycosylated in human plasma. [11] [12].

The latest dialyzer membranes used for hemodialysis have the ability to remove large solutes, like β2-microglobulin. The function of these “super-flux” membranes is to remove larger and protein-bound uremic toxins. Earlier reports showed that plasma BNP and ANP levels are reduced after hemodialysis using a “low-flux” membrane, most likely due to body fluid volume removal and/or dialyzer membrane-mediated removal. However, the effect of super-flux membranes on BNP and BNP-related molecules, such as glycosylated proBNP and glycosylated NT-proBNP, remains unknown. In the present study, therefore, we examined the levels of total BNP, proBNP, mature BNP, glycosylated NT-proBNP, nonglycosylated NT-proBNP, ANP and cGMP in ESRD patients, before and after hemodialysis using a super-flux membrane.

## Subjects and Methods

### Patients

105 healthy subjects and 36 ESRD patients attending routine outpatient hemodialysis sessions were included in the study. Ethical approval was granted by the Kyoto University Hospital Ethical Committee. The aims of study were explained to each participant, and written consent was obtained. All clinical investigations were conducted according to the principles expressed in the Declaration of Helsinki.

In the ESRD group, all the patients underwent regular hemodialysis three times a week. The clinical characteristics of the healthy subjects and ESRD patients in this study are listed in Table 1. In the ESRD group, all the patients were dialyzed using super-flux polysulphonedialyzers: PES-19SEa eco in 4 cases, PES-21SEa eco in 8 cases, PES25SEa eco in 3 cases (Nipro, Tokyo, Japan), PS-15EL in 4 cases, APS18EL in 4 cases, APS-21EL in 11 cases and KF-20C in 2 cases (Asahikasei, Tokyo Japan). The mean dialysis session time was 3.96±0.30 h, and the QB was 209±25.1 ml/h. Cardiac function, heart size and blood pressure were relatively well controlled. Total body fluid volume was estimated using the equation: body weight×0.6 [13]. The percent changes in total body fluid volume were calculated as: body weight change/calculated total body weight before hemodialysis.

**Table 1:** Clinical profiles of the healthy subjects and ESRD patients enrolled in this study.

	Healthy subjects (N = 105)	ESRD patients (N = 36)	p
Age, years	51.3±12.1	64.0±11.7	<0.0001
Sex, m/f	50/55	20/16	0.2663
BMI, kg/m^2^	22.2±3.0	20.2±2.4	0.0005
HD vintage, year	(−)	11.2±8.9	
Before HD			
Systolic BP, mmHg	115.4±17.3	142.2±22.4	<0.0001
Diastolic BP, mmHg	73.9±13.0	71.4±10.8	0.3016
Mean BP, mmHg	87.7±14.0	95.0±13.1	0.0071
After HD			
Systolic BP, mmHg		128.5±19.9	
Diastolic BP, mmHg		69.9±9.4	
Mean BP, mmHg		89.5±11.2	
Inter-dialysis weight gain, kg		2.67±0.92	
CTR,%	44.4±4.6	49.7±5.0	<0.0001
BUN, mg/dl	14.0±3.1	59.3±17.2	<0.0001
Cre, mg/dl	0.76±0.14	10.59±1.27	<0.0001
Alb, g/dl	4.3±0.2	3.60±0.35	<0.0001
Hemoglobin, g/dl	14.0±1.3	10.62±1.15	<0.0001
Ht, %	43.1±3.6	32.1±3.6	<0.0001
Diabetes mellitus	(−)	27.8 (10)	
Anti-hypertensive drug	(−)		
Calcium channel blocker		44.4%(16)	
Alpha blocker		2.8%(1)	
Beta blocker		38.9%(14)	
ACEI		2.8%(1)	
ARB		36.1%(13)	
Nitrate		5.6.%(2)	
Digoxin		2.8%(1)	
Statin		25.0%(9)	
Aspirin		30.6%(11)	
Insulin		2.8%(1)	
Echocardiographic data	Not examined		
IVS thickness, mm		10.8±1.9	
PW thickness, mm		11.35±2.0	
LV mass index, g/m^2^		131.7±37.0	
Left atrium diameter, mm		37.9±5.3	
LV end-diastolic diameter, mm		43.8±5.8	
LV end-systolic diameter, mm		26.6±6.1	
Ejection fraction, %		64.2±11.5	

### Blood Sampling

Venous blood was collected before and immediately after dialysis for measurement of total BNP, proBNP, mature BNP, nonglycoNT-proBNP, glycoNT-proBNP, ANP and cGMP. Blood samples were transferred to chilled glass tubes containing disodium EDTA (1 mg/ml) and aprotinin (500 U/ml) and immediately centrifuged at 4°C, after which the resultant plasma was frozen and stored at −80°C until used. We also measured levels of hemoglobin, serum c-reactive protein, albumin and parathyroid hormone, among others.

### Biochemical Analyses

#### Measurement of plasma ANP, cGMP, total BNP, proBNP, and mature BNP

Plasma ANP levels were measured using a specific immunoradiometric assay, while levels of cGMP were measured using a radioimmunoassay, as previously described.^10^

Plasma total BNP and proBNP levels were measured using an immunochemiluminescent assay, as previously described. [9] All BNP assays, regardless of the source (e.g., Shionogi, Biosite), cross-react with proBNP, because the two antibodies used in the assays recognize epitopes common to BNP and proBNP. For that reason, total BNP means the sum of proBNP plus mature BNP, most of which is BNP[1–32]. In the present study, we measured total BNP and proBNP separately and calculated the mature BNP as follows: mature BNP = total BNP − proBNP. [9].

#### Measurement of plasma glycoNT-proBNP and nonglycoNT-proBNP in HD patients

ProBNP is post-translationally glycosylated to varying degrees in its N-terminal region, at Thr36, Ser37, Ser44, Thr48, Ser53, Thr58 and Thr71. [10] NT-proBNP is similarly glycosylated. The Elecsys proBNP II system (Roche Diagnostics, Germany) is comprised of a capture monoclonal antibody that recognizes NT-proBNP[27–31] and a monoclonal signal antibody that recognizes NT-proBNP[42–46], which contains a glycosylation site at amino acid residue 44. [14] Notably, *O*-linked oligosaccharide attachment inhibits the binding of the signal antibody to NT-proBNP. [15], [16] We therefore postulated that NT-proBNP measured using Elecsys proBNP II is actually only nonglycoNT-proBNP. To measure total NT-proBNP, plasma samples were incubated for 24 h at 37°C with or without a cocktail of deglycosylating enzymes, included *O*-glycosidase (Roche Diagnostics) and neuraminidase (Roche Diagnostics) at final concentrations of 4.25 and 42.5 mU/ml, respectively, as described previously. [10] [14] NT-proBNP levels were then measured using Elecsys proBNP II, after which the glycoNT-proBNP level was calculated as: total NT-proBNP – nonglycoNT-proBNP.

#### Echocardiographic measurements

An experienced echocardiographer without knowledge of the clinical features of the patients performed the echocardiography using a cardiac ultrasound unit (Logic 500 MD; GE Healthcare, England) before hemodialysis in the ESRD group. Left atrial diameter, interventricular thickness, posterior wall thickness, left ventricular end-diastolic diameter and left ventricular end-systolic diameter were all measured. Fractional shortening (FS), left ventricular mass index (LVMI) and left ventricular ejection fraction (LVEF) were calculated using standard formulae according to the recommendations of the American Society of Echocardiography.

#### Gel filtration chromatography

We analyzed immunoreactive proBNP levels in plasma to determine whether it is glycosylated in hemodialysis patients. Eluate lyophilized after extraction on a Sep-Pak C18 column (Waters, Milford, MA, USA) was dissolved in phosphate buffer and incubated with or without a cocktail of deglycosylation enzymes for 24 h at 37°C, as described above. The eluate was then lyophilized again and dissolved in 30% acetonitrile containing 0.1% TFA. The resultant solution was separated by gel filtration high-performance liquid chromatography (HPLC) on a Superdex 75 10/300 GL column (10×300 mm×2, GE Healthcare) using the same buffer at a flow rate of 0.4 mL/min. The column effluent was fractionated every minute into polypropylene tubes containing bovine serum albumin (100 mg), after which each fraction was analyzed using our recently developed total BNP and proBNP immunochemiluminescent assay [9].

### Statistical Analyses

All values are expressed as means±SE. The statistical significance of differences between two groups was evaluated using Fisher’s exact test or paired Student’s t test, as appropriate. The distribution of plasma peptide levels was normalized by log transformation, when appropriate. The statistical significance of differences among three or more groups was evaluated using one-way analysis of variance followed by Bonferroni’s multiple comparison test. Correlation coefficients were calculated using linear regression analysis. Values of p<0.05 were considered significant.

## Results

The clinical profiles of the healthy subjects and ESRD patients enrolled in this study are shown in Table 1. The healthy subjects received no medication. Among the ESRD patients, 86% were prescribed antihypertensive medication (Table 1). The average percentage weight loss during hemodialysis was 4.9% (2.2% to 8.4%), with a post-dialysis relative extracellular fluid volume of 20.2% (11.1% to 31.5%). Standard two-dimensional transthoracic echocardiography revealed an average cardiac ejection fraction of 64% (36% to 92%), and the cardiothoracic ratio measured on posterior-anterior chest X-rays was 50±5%.

### Plasma Concentrations of Total BNP, proBNP, Mature BNP, nonglycoNT-proBNP, glycoNT-proBNP, ANP and cGMP in Healthy Subjects and ESRD Patients Before Hemodialysis

Plasma levels of total BNP, proBNP, mature BNP, nonglycoNT-proBNP, glycoNT-proBNP, ANP and cGMP in healthy subjects and ESRD patients before hemodialysis are shown in Table 2. All seven parameters were significantly higher in the hemodialysis patients than the healthy subjects (p<0.0001). In addition, glycoNT-proBNP levels was significantly higher than nonglycoNT-proBNP in both groups. There was no significant difference in the glycoNT-proBNP/nonglycoNT-proBNP ratio (healthy subject vs. ESRD patients: 4.6±1.8 vs. 4.4±1.7; n.s.) between the two groups.

**Table 2:** Natriuretic peptide-related molecules in healthy subjects and ESRD patients.

	Healthy subjects (n = 105)	ESRD patients (n = 36)	p
Total BNP, pM	1.8±22.0	35.7±34.4	<0.0001
proBNP, pM	1.2±1.2	22.6±22.7	<0.0001
Mature BNP, pM	0.6±0.8	13.1±12.9	<0.0001
Nonglyco-NT-proBNP, pM	44.8±64.5	600.3±779.1	<0.0001
Glyco-NT-proBNP, pM	173.0±157.3	1934.2±1829.5	<0.0001
ANP, pM	21.1±12.4	44.3±30.9	<0.0001
cGMP, nM	2.9±1.3	20.2±13.0	<0.0001

### Differences in the Plasma Concentrations of Total BNP, proBNP, Mature BNP, nonglycoNT-proBNP, glycoNT-proBNP, ANP and cGMP before and after Hemodialysis

**Figure 1:**
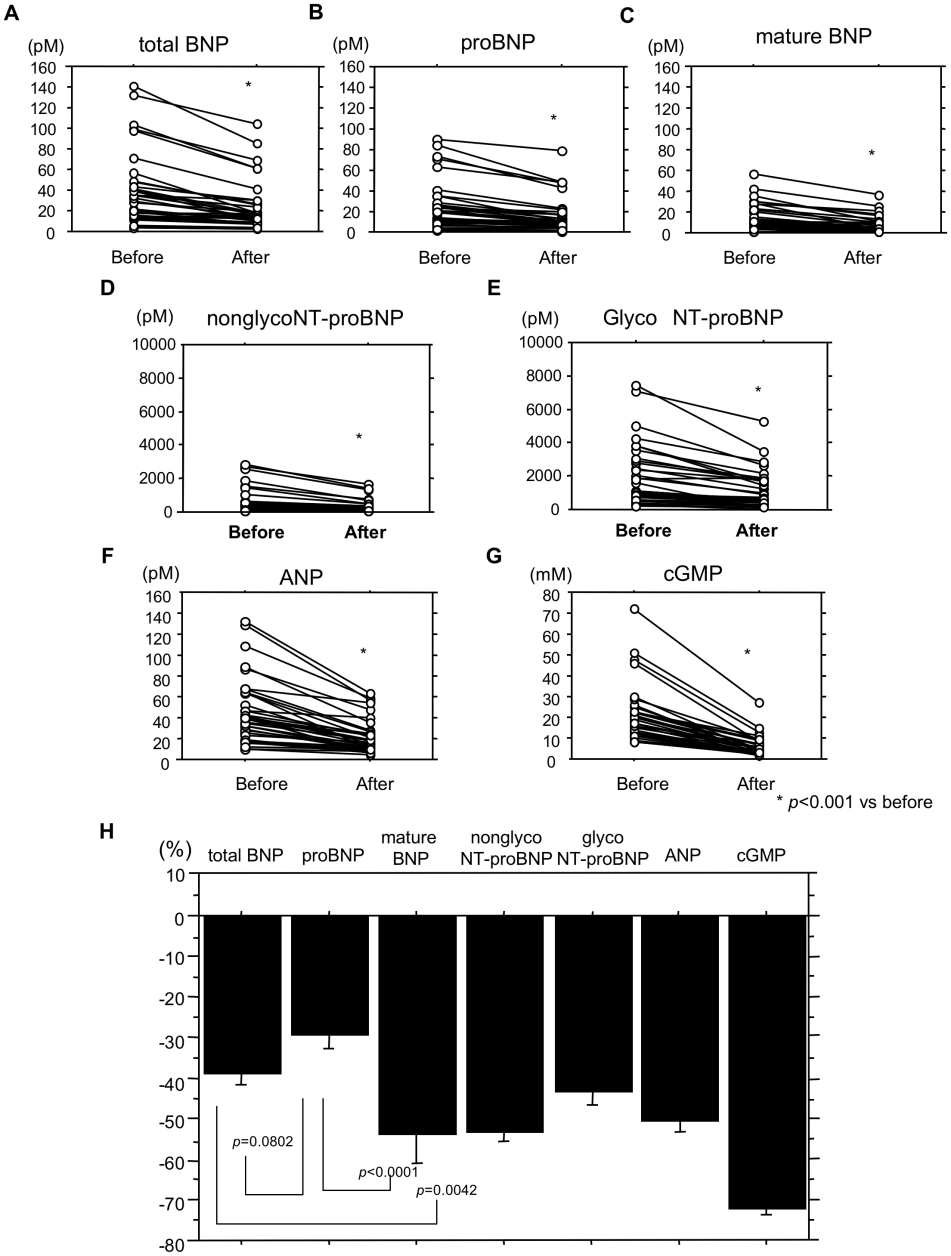
Changes in plasma levels of individual parameters during hemodialysis. (A–G) Individual changes in the levels of total BNP (A), proBNP (B), mature BNP (C), nonglycoNT-proBNP (D), glycoNT-proBNP (E), ANP (F) and cGMP (G) in ESRD patients during hemodialysis. Values are means ± SE. *p<0.001 vs. before hemodialysis. Before, before hemodialysis; After, after hemodialysis. (H) Reduction ratios for total BNP, proBNP, mature BNP, nonglycoNT-proBNP, glycoNT-proBNP, ANP and cGMP in ESRD patients during hemodialysis. Values are means ± SE.

We next focused on the profile of natriuretic peptide-related molecules in ESRD patients. We initially evaluated the changes in plasma levels of total BNP, proBNP, mature BNP, nonglycoNT-proBNP, glycoNT-proBNP, ANP and cGMP associated with hemodialysis. As shown in Figure 1A, B, C, D, E, F and G, levels of all seven parameters were significantly lower after hemodialysis (p<0.001).

As shown in Figure 1H, the reduction ratios were calculated using the formula (A−B)/A, where A and B were the plasma concentrations before and after hemodialysis, and the values obtained give the relative magnitudes of the reductions. The reduction ratio for proBNP was smaller than the others, whereas the ratio for cGMP, which had the smallest molecular weight (MW = 523), was the largest among the molecules tested. The reduction ratio for mature BNP (MW = 3500) was significantly greater than that for proBNP, but was about the same as those for ANP (MW = 3000) and nonglycoNT-proBNP (MW = 8500). And not surprisingly, the reduction ratio for total BNP, which includes proBNP plus mature BNP, was between the ratios for mature BNP and proBNP.

It is well known that decreasing cardiac load reduces circulating levels of BNP and ANP, and that decreasing total body fluid volume could reduce cardiac preload. We therefore evaluated the relationship between dialysis-induced loss of body weight and total body fluid volume and the plasma levels of total BNP, proBNP, nonglycoNT-proBNP, glycoNT-proBNP and ANP. However, we found no significant correlation between the percent body weight change or percent total body fluid volume loss and the percent reduction of any of these peptides (Table 3).

**Table 3:** Correlation coefficients and p values relating changes in percent body weight or total body fluid volume to the percent reduction in natriuretic peptide-related molecules.

	% body weight change		% total body fluid volume change	
% Reduction rate	r	p	r	p
total BNP	−0.086	0.621	−0.096	0.580
mature BNP	−0.130	0.452	−0.124	0.474
proBNP	−0.039	0.823	−0.054	0.756
ANP	−0.117	0.500	0.119	0.493
nonglyco- NT-proBNP	−0.120	0.488	−0.122	0.483
glyco- NT-proBNP	−0.208	0.224	−0.209	0.222
cGMP	−0.047	0.785	−0.034	0.846

r is the correlation coefficient.

### Gel-filtration Chromatography Before and after Deglycosylation Procedure

**Figure 2:**
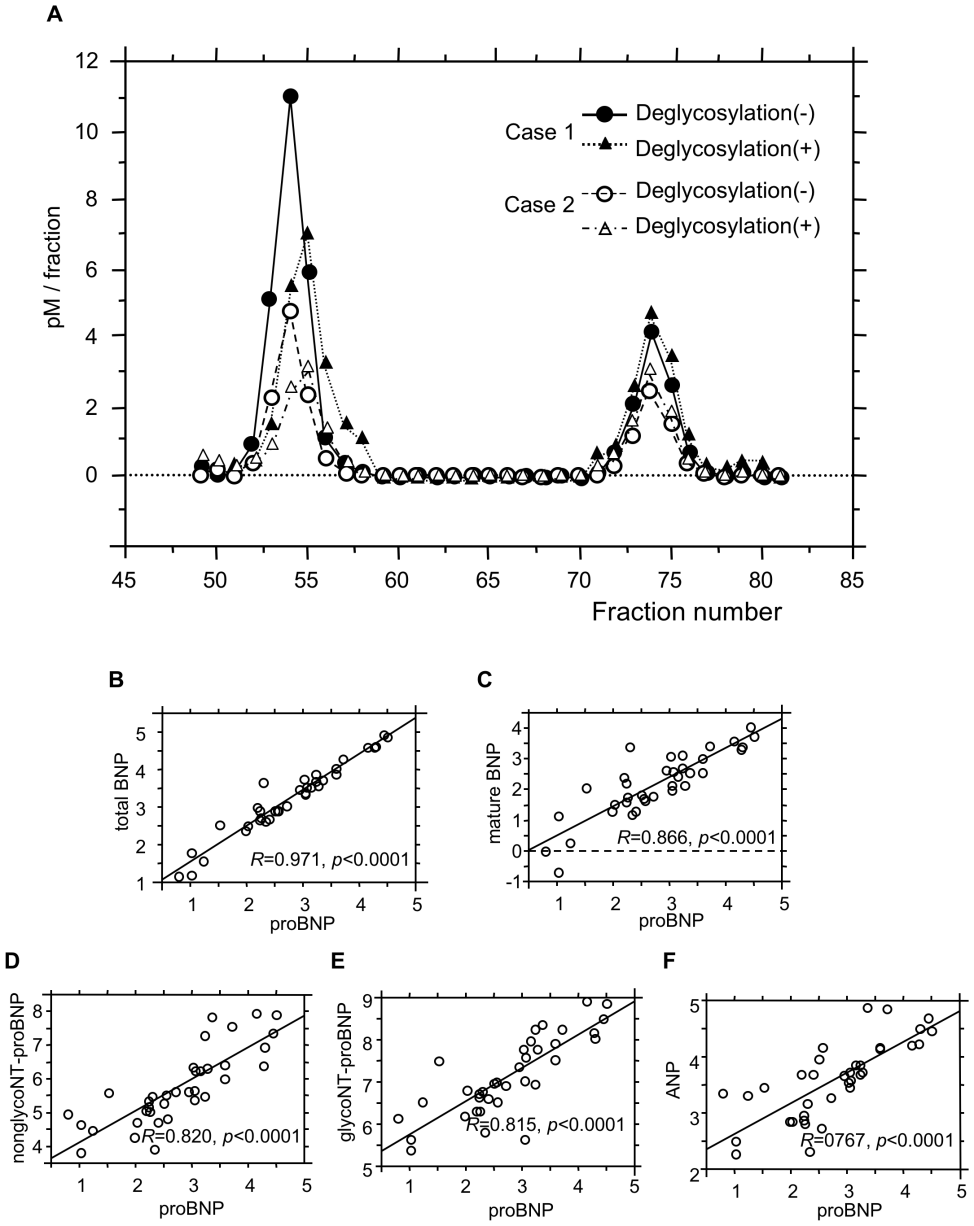
Gel filtration analysis of total BNP and proBNP in hemodialysis patients. (A) Fractions were assayed using total BNP systems. The first and second peaks show proBNP and BNP-32, respectively. (B–F) Correlations between proBNP and other natriuretic peptide-related molecules in ESRD patients before hemodialysis. *R* is the correlation coefficient. Values are expressed as log_10_ of each level of the indicated molecules.

Figure 2A shows two immunoreactive BNP peaks detected using the total BNP assay with gel filtration fractions. The first peak appeared in fractions 52–55 and the second peak in fractions 72–75. When subjected to gel filtration, recombinant proBNP, glycosylated proBNP and BNP were eluted mainly in fractions 53, 56 and 74, respectively. Treating the same plasma sample with an enzyme cocktail catalyzing deglycosylation shifted the first peak to fractions 54–56, which is consistent with the proBNP peak. These results suggest the major molecular form of proBNP in the plasma of hemodialysis patients is glycosylated proBNP.

### Relationship between proBNP and Total BNP, Mature BNP, nonglycoNT-proBNP, glycoNT-proBNP and ANP

We next evaluated the relationships between proBNP and the other natriuretic peptide molecules in hemodialysis patients (Figure 2B, C, D, E and F). We found that levels of proBNP significantly correlated with those of total BNP, mature BNP, nonglycoNT-proBNP, glycoNT-proBNP and ANP, most of which are established biomarkers.

### Relationship between cGMP and Total BNP, proBNP, Mature BNP, nonglycoNT-proBNP, glycoNT-proBNP and ANP in ESRD Patients before and after Hemodialysis

**Figure 3:**
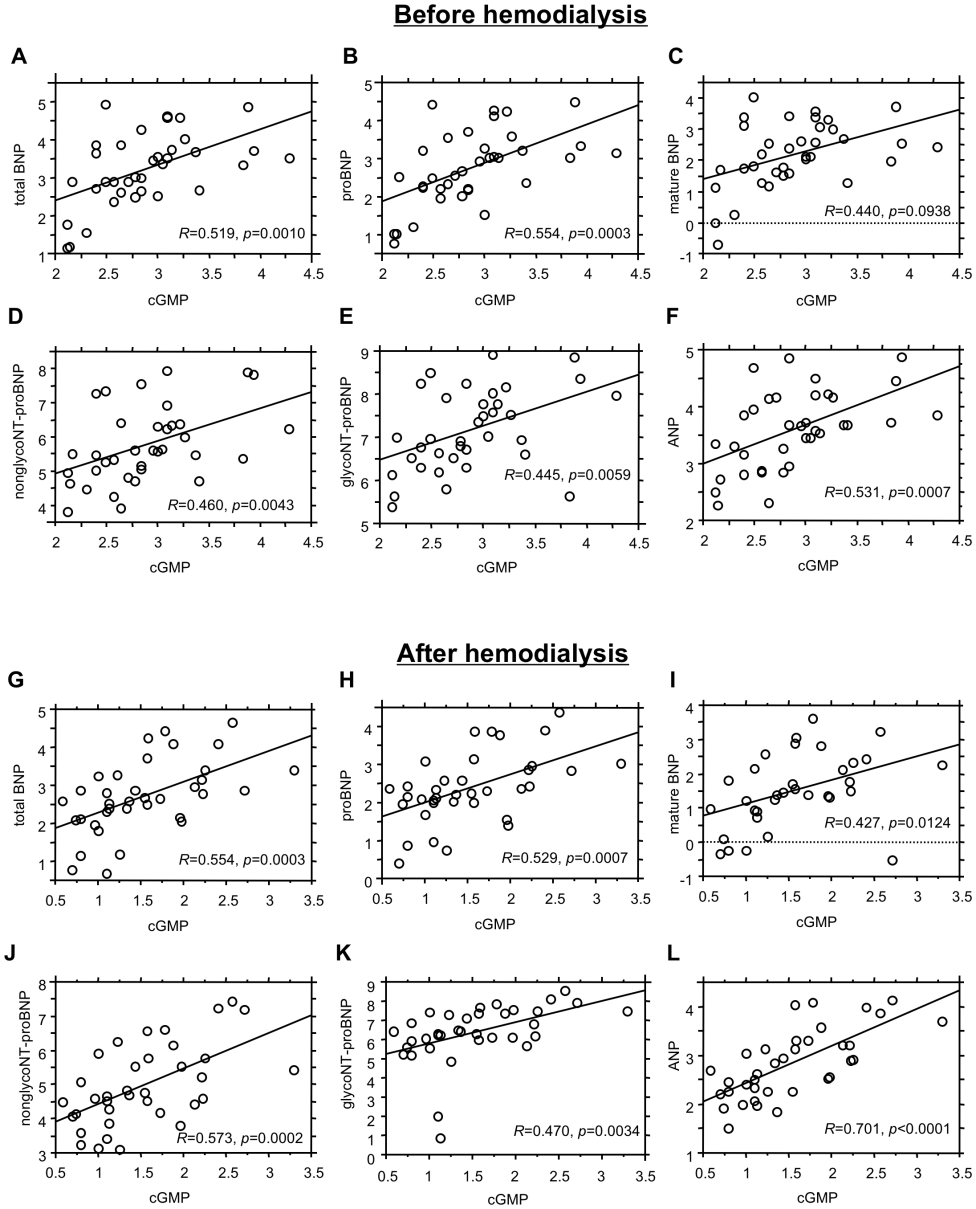
Correlations between cGMP and natriuretic peptide-related molecules in ESRD patients before and after hemodialysis. *R* is the correlation coefficient. Values are expressed as log_10_ of each level of the indicated molecules.

When we evaluated the correlation between the levels of cGMP and those of natriuretic peptide-related molecules, we found that levels of total BNP, proBNP, mature BNP, nonglycoNT-proBNP, glycoNT-proBNP and ANP all correlated significantly with cGMP, both before and after hemodialysis (Figure 3). In particular, proBNP appeared to correlate more strongly with cGMP before hemodialysis than did the other molecules. After hemodialysis, ANP showed the highest correlation with cGMP among the natriuretic peptide-related molecules.

### ProBNP/Total BNP Ratios, Biochemical Parameters and Patient Profiles

**Figure 4:**
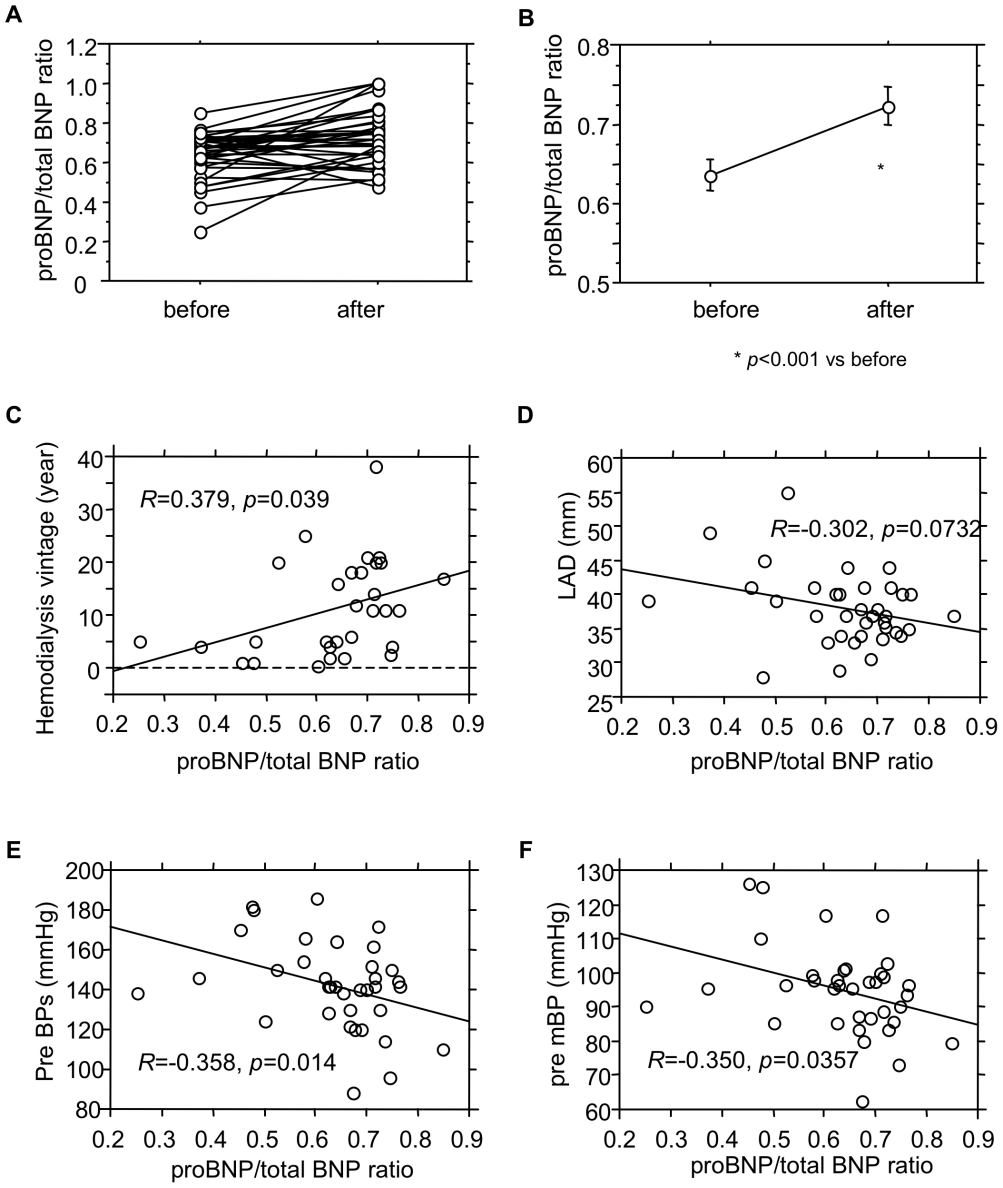
ProBNP/total BNP ratios and their correlation with the indicated clinical parameters. (A) Individual changes in proBNP/total BNP ratios in ESRD patients during hemodialysis: Before, before hemodialysis; After, after hemodialysis. (B) ProBNP/total BNP ratios in ESRD patients during hemodialysis expressed as means ± SE. (C–F) Correlation between proBNP/total BN ratios and hemodialysis vintage (C), left atrial diameter (LAD) (D), systolic blood pressure before hemodialysis (preBPs) (E) and mean blood pressure before hemodialysis (pre mBP) (F). *R* is the correlation coefficient between the indicated parameters.

It was previously suggested that the ratio of proBNP to total BNP varied widely, depending on the patient’s heart failure status. [8] We next evaluated proBNP/total BNP ratios in ESRD patients. As shown in Figure 4, ProBNP/total BNP ratios were significantly increased after hemodialysis, which could, in part, reflect the fact that the relative reduction in proBNP was smaller than that for total BNP. There was also a significant (but weak) positive correlation with hemodialysis vintage. Upon examination of the patient profiles, we found no significant correlation between the proBNP/total BNP ratios and any other biochemical parameter. ProBNP/total BNP ratios showed weak but significant negative correlations with systolic and mean blood pressures (R = −0.358, P = 0.014, R = −0.350, P = 0.036), and tended toward a negative correlation with left atrial diameter (R = −0.302, p = 0.073).

### NonglycoNT-proBNP and glycoNT-proBNP in Hemodialysis Patients

**Figure 5:**
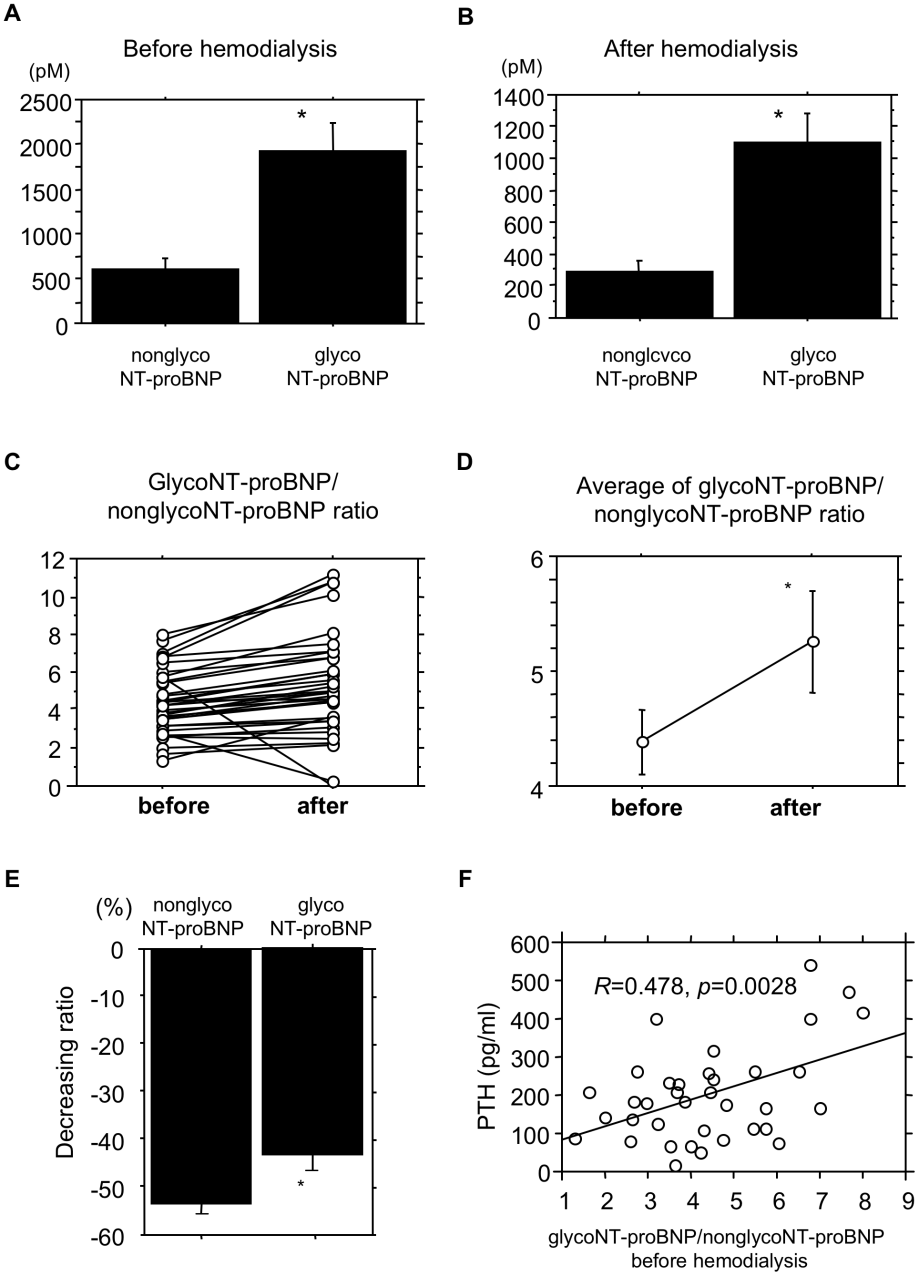
NonglycoNT-proBNP and glycoproBNP levels, glycoNT-proBNP/nonglycoNT-proBNP ratios and correlations between glycoNT-proBNP/nonglycoNT-proBNP ratios and clinical parameters. (A, B) Circulating levels of nonglycoNT-proBNP and glycoNT-proBNP in ESRD patients before (A) and after (B) hemodialysis. Values are expressed means ± SE. (C, D) Individual changes and values of glycoNT-proBNP/nonglycoNT-proBNP ratios in ESRD patients during hemodialysis. Values are expressed as mean ± SE. *p<0.001 vs. before hemodialysis. before, before hemodialysis; after, after hemodialysis. (E) Reduction ratios for nonglycoNT-proBNP and glycoNT-proBNP during hemodialysis. *p<0.001 vs. nonglycoNT-proBNP. (F) Correlation between the glycoNT-proBNP/nonglycoNT-proBNP ratio and the serum parathyroid hormone (PTH) levels. *R* is the correlation coefficient.

We also compared the levels of glycoNT-proBNP with those of nonglycoNT-proBNP in hemodialysis patients. We found that levels of glycoNT-proBNP were several times higher than those of nonglycoNT-proBNP in the patients before hemodialysis; nonetheless, the glycoNT-proBNP/nonglycoNT-proBNP ratio was significantly larger after hemodialysis than before it (Figure 5). This is in part because the relative reduction in nonglycoNT-proBNP during hemodialysis was significantly greater than that for glycoNT-proBNP. We then evaluated the correlations between the glycoNT-proBNP/nonglycoNT-proBNP ratios and other biochemical parameters, which revealed the ratio correlated significantly with serum parathyroid hormone levels in the patients, but not with serum calcium or phosphate levels (data not shown).

## Discussion

Plasma BNP and NT-proBNP levels are elevated in patients with heart failure, [17] [18] [19] correlate strongly with LV filling pressure, and increase with increasing severity of heart failure evaluated based on New York Heart Association Class [20] [21], functional capacity [22], or systolic and diastolic dysfunction [23], [24]. Even in patients with chronic kidney disease and ESRD, left ventricular end-diastolic wall stress remains a strong determinant of circulating BNP levels [25]. Moreover, in ESRD patients receiving long-term dialysis, BNP and NT-proBNP levels are strongly associated with the severity of LV hypertrophy and systolic dysfunction [6] [26], [27], [28], [29], [30], and their elevation also reflects the presence of myocardial ischemia and is indicative of the severity of coronary artery disease [31]. Finally, BNP and NT-proBNP are highly predictive of subsequent cardiac events and mortality in hemodialysis patients. [32].

It was once thought that proBNP is cleaved to BNP and NT-proBNP within cardiac myocytes and then secreted into the circulation. However, recent studies have shown that circulating levels of unprocessed precursor proBNP are elevated in heart failure. In addition, proBNP and NT-proBNP contain seven sites suitable for *O*-linked oligosaccharide attachment within their N-terminal regions. In the present study, therefore, we measured plasma levels of proBNP, total BNP, mature BNP, nonglycoNT-proBNP, glycoNT-proBNP, ANP and cGMP, in healthy subjects and ESRD patients, and found that all of them were higher in ESRD patients, most of whom had preserved cardiac systolic function (Table 1), than in healthy persons. These increases in ESRD patients can be explained by reductions in their clearance due to the renal failure, increased plasma volume, and diastolic dysfunction caused by left ventricular hypertrophy, among others. Moreover we also found that all of these molecules declined during hemodialysis, though the magnitudes of the reductions did not correlate with any indices of body fluid volume. These results may be explained in part by dialyzer membrane-mediated removal. The so-called super-flux membranes currently in use are able to remove medium-sized molecular solutes, such as β2-microglobulin (MW = 12,000), which enables removal of larger and protein-bound uremic toxins. The pore size for the membranes used in the present study was 78–84 Å, and the sieving coefficient for β2-microglobulin was 0.99. It is therefore likely that the super-flux membranes removed a great deal of BNP and BNP-related molecules during hemodialysis.

Consistent with that idea, the reduction ratio for cGMP, which has a molecular weight of about 500, was the largest, while the reduction ratio for proBNP, which has a molecular weight of about 20,000∼25,000, was the smallest. The reduction ratio for mature BNP (MW: 3,500) was similar to that of ANP (MW 3,000), reflecting the similarity of their molecular weights. The reduction ratio for total BNP was comparatively modest, because it is the mean of the ratios for mature BNP and proBNP. It thus appears that the magnitudes of the reductions in peptide concentration associated with hemodialysis depends on their molecular weights.

Previously, we also reported the plasma levels of BNP, nonglycoNT-proBNP, glycoNT-proBNP, ANP and cGMP in patients with chronic renal failure before and after hemodialysis. [14] In that study, patients were hemodialyzed using older generation dialyzers that removed less of the large solutes like β2-microglobulin than the super-flux membrane we used in the present study. In that earlier study the calculated reduction ratio for each parameter during hemodialysis was as follows: BNP 37.9%, nonglycoNT-proBNP 23.0%, glycoNT-proBNP 4.4%, ANP 61.0%, and cGMP 75.0%. The reduction ratio for nonglycoNT-proBNP and glycoNT-proBNP, which are higher molecular weight molecules, was much smaller than in the present study, most likely due to the difference in the membrane’s ability to remove large molecules.

In the present study, we used the Elecsys proBNP II assay (Roche Diagnostics, Indianapolis, IN, USA) to measure nonglycoNT-proBNP and glycoNT-proBNP, with and without deglycosylation enzyme treatment. This assay is the most frequently used NT-proBNP assay in the world and contains a capture monoclonal and a signal monoclonal antibody that recognizes NT-proBNP[27–31] and NT-proBNP[42–46], respectively. Notably, NT-proBNP[42–46] has a glycosylation site at amino acid 44, and a recent study demonstrated that *O*-linked oligosaccharide attachment markedly inhibits binding of the antibody to its antigen peptide [16]. Consequently, Elecsys proBNP II is thought to measure only nonglycoNT-proBNP. We found that nonglycoNT-proBNP was greatly elevated in ESRD patients and that levels of glycoNT-proBNP were 4–5 times higher than those of nonglycoNT-proBNP. The remarkable increase of nonglycoNT-proBNP in hemodialysis patients is thought to reflect the following conditions: (1) NT-proBNP does not bind to the natriuretic peptide receptor-A or -C; (2) NT-proBNP is not metabolized by neutral endopeptidase; and (3) clearance of NT-proBNP is largely dependent on excretion from the kidney. The glycoNT-proBNP/nonglycoNT-proBNP ratio was larger after hemodialysis than before it. This is most likely because the molecular weight of glycoNT-proBNP is much larger than that of nonglycoNT-proBNP, so it is less likely to be affected by membrane-dependent removal than nonglycoNT-proBNP.

Using gel-filtration HPLC combined with direct chemiluminescent immunoassay, in the present study we showed that the major molecular form of proBNP in the plasma of hemodialysis patients is glycosylated proBNP. Similarly, recent studies have also shown that the major molecular form of plasma proBNP in patients with heart failure and control is glycosylated proBNP [9]. It is thus possible that proBNP in human plasma is glycosylated. We found that proBNP levels were closely correlated with those of total BNP, mature BNP, nonglycoNT-proBNP, glycoNT-proBNP and ANP. This suggests proBNP may also be a useful biomarker of cardiovascular disease, like BNP and NT-proBNP, two well-established biomarkers of cardiovascular disease in ESRD patients. In addition, levels of proBNP correlated significantly with cGMP before hemodialysis, suggesting proBNP may be a good marker of the biological action of natriuretic peptides. After hemodialysis, proBNP still correlated significantly with cGMP, but the correlation coefficient was modest. This may be because the reduction ratio for proBNP is small, while that for cGMP is large.

Interestingly, the proBNP/total BNP ratio correlated positively with hemodialysis vintage and correlated negatively with LAD and blood pressure. Longer hemodialysis vintage may lead to accumulation of the glycosylated proBNP, and diastolic dysfunction and/or increased afterload may alter the processing of glycosylated proBNP to BNP and glycoNT-proBNP. Notably, the glycoNT-proBNP/nonglycoNT-proBNP ratio correlated positively with parathyroid hormone levels in hemodialysis patients. Given that parathyroid hormone induces cardiac hypertrophy via its receptor on myocytes [33], its signal may influence glycosylation within cardiac myocytes and thus the glycoNT-proBNP/nonglycoNT-proBNP ratio. Further studies will be needed to elucidate the mechanism involved.

It has been suggested that hemodialysis reduces natriuretic peptide levels through dialytic clearance or by improving volume control, which results in decreased cardiac overload and reduced secretion from the heart [34]. A limitation of the present study is that we evaluated changes in volume status during hemodialysis based only on changes in body weight or estimated total body fluid volume. No other methods (e.g., bioimpedance analysis) were used. In addition, we did not perform echocardiography after hemodialysis. Had we evaluated changes in cardiac load during hemodialysis using different methods, perhaps some relationship might have been found between changes of the levels of natriuretic peptide-related molecules during hemodialysis and the magnitude of the reduction in cardiac load. Nonetheless, our results suggest that the reduction in the natriuretic peptide-related molecules after hemodialysis is due not only to the reduction in atrial overload, but also to removal via the super-flux dialyzer.

In conclusion, this is a first report showing the hemodialysis-associated changes of natriuretic peptide-related molecules, including proBNP, in ESRD patients in the super-flux dialyzer era. With the development of the super-flux dialysis membrane, there has been a marked change in the kinetics of molecules before and after hemodialysis. In addition, recent studies have shown that glycosylated proBNP is a major molecular form in human plasma and that glycosylated NT-proBNP is underestimated by the NT-proBNP assay system currently being used. Under these conditions, correct interpretation of the peptide levels in the plasma of ESRD patients undergoing hemodialysis and their clinical application may require careful consideration. Further study will be necessary to determine which BNP-related peptides, including proBNP, are most indicative of cardiac complications and predictive of prognosis in hemodialysis patients.
